# Development of Agranulocytosis after Discontinuation of Methimazole: An Unusual Case

**DOI:** 10.1155/2015/974524

**Published:** 2015-08-03

**Authors:** Rıfkı Üçler, Murat Atmaca, Ömer Candar, Murat Alay, Burhan Göy, Erdal Kara, Mahfuz Turan, Yusuf Demir

**Affiliations:** ^1^Department of Endocrinology and Metabolism, Yuzuncu Yil University Faculty of Medicine, 65080 Van, Turkey; ^2^Department of Internal Medicine, Yuzuncu Yil University Faculty of Medicine, 65080 Van, Turkey; ^3^Department of Internal Medicine, Bulanık State Hospital, 49500 Mus, Turkey; ^4^Department of Hematology, Yuzuncu Yil University Faculty of Medicine, 65080 Van, Turkey; ^5^Department of Otorhinolaryngology, Yuzuncu Yil University Faculty of Medicine, 65080 Van, Turkey; ^6^Department of Nuclear Medicine, Yuzuncu Yil University Faculty of Medicine, 65080 Van, Turkey

## Abstract

Agranulocytosis is a rare and critical adverse effect of antithyroid drugs (ATD). The occurrence of agranulocytosis in continuous ATD treatment patients is well known; however, a case of ATD agranulocytosis occurring following the discontinuation of methimazole (MMI) treatment is not a usual situation. We herein describe a case of a 41-year-old woman who was previously administered methimazole (MMI) for ten days and developed ATD-induced agranulocytosis and symptoms of an upper respiratory tract infection after three weeks following discontinuation of MMI treatment. A thorough hematologic and serological evaluation did not disclose an alternative cause for the agranulocytosis. After receiving empirical antibiotic treatment, she responded successfully with clinical improvement of her symptoms and resolved neutropenia on the seventh day. This case is atypical because agranulocytosis developed after discontinuation of MMI, which strengthens the importance of remaining alert for signs of agranulocytosis even after discontinuation of ATD treatment.

## 1. Introduction

Antithyroid drug- (ATD-) induced agranulocytosis is a rare but potentially life threatening toxicity, developing in only 0.1–1.0% of patients taking ATD [1]. Agranulocytosis generally occurs within the first 2-3 months of treatment [2]; however, some cases have indicated that agranulocytosis may occur following long-term treatment [3]. The occurrence of agranulocytosis in continuous ATD treatment patients is well known; however, a case of ATD agranulocytosis occurring following the discontinuation of methimazole (MMI) treatment is not a usual situation. We present a case of agranulocytosis that occurred three weeks after the discontinuation of MMI treatment.

## 2. Case Presentation

A 41-year-old female presenting with complaints of fever, chills, and symptoms of an upper respiratory tract infection including sore throat and difficulty swallowing for three days arrived at our emergency department in December 2014. The patient did not have vomiting, diarrhea, chest distress, abdominal pain, rash, jaundice, cough, or expectoration. The patient had no history of smoking or alcohol intake, hypertension, diabetes, kidney disease or other chronic diseases, tuberculosis, hepatitis, typhoid fever, or other infectious diseases. In addition, there was no history of trauma, surgery or history of medicine, or food allergies. One month previously, the patient had been diagnosed with hyperthyroidism and prescribed methimazole (10 mg BID) and propranolol (20 mg BID) at a local hospital. The patient discontinued the medicine after taking this treatment for ten days and three weeks prior to admission, since she thought that she may be pregnant because of a delayed menstrual cycle.

The patient's initial assessment in the emergency department revealed a temperature of 39.9°C, heart rate of 129 beats/min, and blood pressure of 174/76 mmHg. The patient had pharyngeal congestion, bilateral tonsil enlargement, and visible purulent secretions on bilateral tonsils. The patient had a mild hand tremor; however, there was no rash or jaundice. There were normal S1 and S2 sounds in cardiac examination. The patient's abdomen was normal, and no peripheral edema or lymphadenopathy was observed. There was bilateral grade 2 thyroid enlargement. There was no evidence of ophthalmopathy. The results of a neurologic and rheumatic examination were unremarkable.

The laboratory findings before antithyroid treatment were as follows: white blood cell count (WBC) and absolute neutrophil count were normal at 5200/*μ*L (reference range, 3500 to 10500/*μ*L) and 2300x/*μ*L (reference range, 1500 to 8000/*μ*L), respectively, with free T3, 16.8 pg/mL (reference range, 1.71–3.71 pg/mL), free T4 3.9 ng/dL (reference range, 0.7–1.48 ng/dL), and thyroid-stimulating hormone (TSH) 0.0001 *μ*IU/mL (reference range, 0.35–4.94 *μ*IU/mL) in thyroid function tests.

The results from peripheral-blood count tests showed an absolute neutrophil count of zero and a WBC count of 400/*μ*L at admission. Hemoglobin concentration and platelet count were normal at 13.6 g/dL (reference range, 11.5–16.5 g/dL) and 165 × 10^3^/*μ*L (reference range, 100–400 × 10^3^/*μ*L), respectively. C-reactive protein level was markedly elevated at 155 mg/L (reference range, 0–5 mg/L). Erythrocyte sedimentation rate was 18 mm/h (reference range, 0–10 mm/h). Thyroid function tests were as follows: free T3, 3.63 pg/mL (reference range, 1.71–3.71 pg/mL), free T4 2.2 ng/dL (reference range, 0.7–1.48 ng/dL), and TSH, 0.0001 *μ*IU/mL (reference range, 0.35–4.94 *μ*IU/mL). The pregnancy test was negative. Results from the electrocardiogram showed sinus tachycardia. Chest radiography divulged no infiltration. An abdominal ultrasonography was unremarkable. A peripheral-blood smear was normal except for agranulocytosis. There were no pathological findings except for borderline anti-nuclear antibody (ANA) positivity in rheumatic, viral, and bacterial serological tests (Table 1). Based on these results, the patient was diagnosed with ATD-induced agranulocytosis, acute tonsillitis, and hyperthyroidism.

On admission to our hospital, the patient was given intravenous antibiotics (amikacin and imipenem) to control infection and propranolol to hinder T4 transformation into T3 and to prevent excitatory effects on the heart. Methimazole was not restarted because of the neutropenia. After three days the neutrophil count increased to 600/*μ*L, after six days the neutrophil count increased to 700/*μ*L, and after seven days the neutrophil count increased to 2600/*μ*L. After 10 days of treatment, a blood culture yielded no microbes, the neutrophil count was increased to 2900/*μ*L, and the patient's symptoms were generally improved; the patient no longer had fever or throat symptoms. With clinical improvement of her symptoms and recovered neutropenia, antibiotherapy was discontinued.

Results from the thyroid ultrasonography showed bilateral parenchymal heterogeneity, increment blood supply, and a 16 mm solitary nodule in the right thyroid lobe. On thyroid scintigraphy, there was diffusely increased radioiodine uptake in the thyroid gland and a hypoactive nodule in the right lobe. Fine needle aspiration biopsy performed at two different times on the nodule was insufficient. The option of surgical treatment was agreed upon because possible thyroid malignancy could not be ruled out due to inadequate biopsy results. In January 2015, she received a bilateral total thyroidectomy for surgical treatment after obtaining euthyroidism with combination therapy including lugol solution, corticosteroid, cholestyramine, and propranolol. The results of surgical histopathology were consistent with a benign nodular hyperplasia. The patient was followed with levothyroxine replacement treatment and the absolute neutrophil count remained within the reference range at 1600 to 3600/*μ*L.

## 3. Discussion

The incidence of ATD-induced agranulocytosis is rare, generally occurring within 2 to 3 months with ATD treatment. In previous reports, except for one case [4], it occurred following continuous ATD treatment [2, 3, 5]. In the present study, a case of ATD-induced agranulocytosis is presented that was seen following treatment with MMI, even though this had been discontinued for three weeks. Our case is the second MMI-induced agranulocytosis case reported in the literature that developed despite discontinuation of MMI treatment.

The pathogenesis of ATD-induced agranulocytosis is not completely understood. Various mechanisms for ATD-induced agranulocytosis have been determined. In the case of an antithyroid drug, the agent acts as a hapten and triggers antineutrophil antibody formation and complement fixation, resulting in neutrophil destruction [6]. Toxic effects require between 20 and 40 days of exposure, and the onset is insidious. It is usually dose- and concentration-dependent and is associated with continuous administration. The antithyroid drug must be present for neutrophil destruction [7]. However, the present report is not in coherence with these mechanisms. In addition, cases of agranulocytosis occurring with 30–90 days of antithyroid therapy have been noticed in the literature [5]. Due to these aforementioned reasons, our case is very exceptional because agranulocytosis was only developed after a short time of ten days. Moreover, ATD-induced agranulocytosis after three weeks following discontinuation of MMI treatment is a most unusual and unexpected situation since it occurred following continuous ATD treatment [2, 3, 5]. It is obvious that the mechanism for the neutropenia in our patient was not dependent on the presence of drug, since the serum half-life of MMI is four to six hours and MMI is almost fully cleared from the blood within 48 hours after oral administration [8]. We believe that late destruction of neutrophils due to an earlier onset immune interaction may be present in our case.

The first case of agranulocytosis in a 27-year-old female despite discontinuation of MMI treatment was reported in 2014 in the literature. In this case report [4], due to relapse of disease after 2 years of methimazole therapy, retreatment of methimazole therapy with 30 mg/day was started and continued for 2 months. Further, agranulocytosis developed after four months following discontinuation of MMI treatment. In our case, as different from the mentioned case, methimazole was firstly started as a 20 mg/day. In our case, both the total duration of antithyroid therapy (10 days) and the period of development of agranulocytosis (3 weeks) were shorter than their case. Granulocyte-colony stimulating factor (GCSF) was administered to raise neutrophil numbers, and after 10 days of treatment, the neutrophil count was improved in their case. In our case agranulocytosis completely resolved on the seventh day although it had not been given GCSF. Results from marrow biopsy showed hyperplasia with karyocyte proliferation and granulocyte precursors normal in this previously reported case. This previous case report did not contain information on rheumatic, viral, or bacterial serological tests, which may cause agranulocytosis, although they made detailed hematological investigations. In our case report, these serological tests were performed following hematology consultation and no pathological findings were determined except for borderline ANA positivity (Table 1). This positivity probably depended on an increased prevalence of anti-nuclear autoantibodies from autoimmune thyroid disease [9], because of the lack of evidence pointing to any rheumatic disease in the examinations or evidence of systemic lupus erythematosus, Sjögren syndrome, or other rheumatic diseases. Additionally, a peripheral-blood smear was normal except for agranulocytosis in our case. However, investigations of bone marrow aspiration and/or biopsy were not performed in our case, since there were no clinical or laboratory findings, such as LDH increase, calcium, phosphorus, or uric acid abnormalities, hepatosplenomegaly, lymphadenopathy, or bone pain, which could arouse suspicion of hematologic malignancy, and also the neutropenia completely recovered in a short time of ten days.

In conclusion, awareness of the possibility of the development of agranulocytosis remains significant, even after several weeks or maybe even after months following discontinuation of ATD-therapy. Thus, this case illustrates the importance of remaining vigilant for signs of agranulocytosis even after stopping ATD treatment, especially with MMI.

## Figures and Tables

**Table 1:** Biochemical and serological test results of the case.

Bacterial serological tests	
*Brucella agglutination test *	*Negative *
*Salmonella typhi O *	*Negative *
Viral serological tests;	
*EBV VCA IgM *	*Negative *
*Parvovirus B19 IgM *	*Negative *
*Anti-CMV IgM *	*Negative *
*Anti-rubella IgM *	*Negative *
*Anti-toxoplasma IgM *	*Negative *
Rheumatic serological tests;	
*ANCA *	*Negative *
*ANA *	***Borderline positive***
*anti-dsDNA *	*Negative *
*anti-ENA *	*Negative *
Blood biochemistry;	
AST	22 U/L
ALT	26 U/L
LDH	321 U/L
Cre	0.63 mg/dL
Uric acid	4 mg/dL
T. bil	0.79 mg/dL
T. prot	5.3 g/dL
Alb	2.8 g/dL
Na	140 mmol/L
K	3.6 mmol/L

EBV: Epstein-Barr virus, VCA: viral capsid antigen, CMV: cytomegalovirus, ANCA: anti-neutrophil cytoplasmic antibodies, ANA: anti-nuclear antibody, dsDNA: double stranded DNA, and ENA: extractable nuclear antigens.
